# Specialty-specific Evaluation of Virtual care Outcomes: A retrospective QUality and safety analysis (S-EVOQUe)

**DOI:** 10.1371/journal.pdig.0000708

**Published:** 2025-01-29

**Authors:** Shawn Mondoux, Frank Battaglia, Anastasia Gayowsky, Natasha Clayton, Cailin Langmann, Paul Miller, Alim Pardhan, Julie Mathews, Alex Drossos, Keerat Grewal

**Affiliations:** 1 Department of Medicine, Division of Emergency Medicine, McMaster University, Hamilton, Ontario, Canada; 2 Institute of Health Policy, Management and Evaluation, Dalla Lana School of Public Health, University of Toronto, Toronto, Ontario, Canada; 3 St. Joseph’s Healthcare Hamilton, Hamilton, Ontario, Canada; 4 ICES, Toronto, Ontario, Canada; 5 Hamilton Health Sciences, Hamilton, Ontario, Canada; 6 Department of Psychiatry and Behavioral Neurosciences, McMaster University, Hamilton, Ontario, Canada; 7 Schwartz/Reisman Emergency Medicine Institute, Sinai Health, Toronto, Ontario, Canada; 8 Division of Emergency Medicine, Department of Medicine, University of Toronto, Toronto, Ontario, Canada; Purdue University, UNITED STATES OF AMERICA

## Abstract

The objective was to compare specialty-specific 7- and 30-day outcomes between virtual care visits and in-person visits which occurred during the SARS-CoV-2 pandemic. Using administrative data from provincial databases in Ontario, ambulatory care visits occurring virtually and in-person during specific timeframes within the pandemic were analyzed. Virtual care visits were matched with corresponding in-person visits based on multiple baseline patient characteristics. We assessed short-term patient outcomes at 7 and 30 days, including subsequent visits, hospital and ICU admissions, surgeries, and mortality and compared them using multivariate logistic regression. Odds ratios were calculated as measures of association between populations. For statistical significance, we used 99% confidence intervals to account for the increased likelihood of chance findings due to the multiple comparisons conducted. Overall, 9,340,519 visits were compared between populations using a 1:1 match on a 20% random sample of the available eligible visits. Over 70% of patients included were seen by a General Practitioner. With few exceptions and across almost all specialties, revisits, ED visits, admissions, ICU and OR use, and mortality were found to be more frequent for patients seen in person. When using the administrative data available to policy makers, there is no evidence to suggest that, in the short-term, virtual care is less safe than in person care. The causes for worse in-person outcomes are not yet clear although are likely related to the streaming of more acutely unwell patients towards in-person care.

## Author summary

Following the declaration of the SARS-CoV-2 pandemic, virtual care was rapidly adopted to address patient and provider needs while reducing the risk of contagion. However, the impact of this swift transition remains understudied, and it is unclear which patients, conditions, and medical specialties are best suited for virtual care. In this study, we analyzed 18 million patient visits in Ontario, Canada, comparing outcomes between in-person and virtual care. Focusing on the medical specialty of the treating physician, we assessed how often patients required urgent or emergent care, surgery, hospitalization, or experienced death within 7 and 30 days of their visit. Our findings showed that, across nearly all specialties, patients seen in person had worse outcomes and were more likely to need additional care than those seen virtually. This likely reflects a tendency for sicker patients to be directed to in-person care, even without formal system guidance. Overall, virtual care patients had positive outcomes, with no specialty showing increased risk of harm. Future research should aim to identify and standardize acuity criteria to guide and expand virtual care services effectively."

## Introduction

Virtual care has rapidly emerged as an integral component of contemporary medical practice. Initially conceived as a solution to overcome geographical barriers [1] its significance escalated exponentially during the COVID-19 pandemic, enabling the continuous delivery of healthcare services amidst social distancing mandates [2]. Consequently, virtual care has swiftly evolved into a normalized care alternative for both patients and healthcare providers. Nonetheless, its widespread adoption necessitates a comprehensive evaluation of its short-term and long-term safety profiles to ensure the absence of any potential risks to patients. The critical examination of this diagnostic and care modality is imperative to prevent the perpetuation of embedded biases, both in terms of care outcomes and public policy, as we forge ahead.

Our previous research [3] has shed light on differences in outcomes between in-person and virtual care during the SARS-CoV-2 pandemic. Additional research into physician specialty-specific outcomes was required to ensure that results were not driven by a subset of specialty groupings. Although our previous study suggests that higher acuity patients may be streamed towards in-person care, it is possible that a specialty-specific analysis might provide more insight into these findings. Although ambulatory visits only rarely are provided with an acuity rating (in the ED environment), this analysis may allow for the analysis of the systematic application of a pragmatic gestalt in patient triage.

Furthermore, examining disparities in specialty-specific outcomes may provide insights into whether specific patient subgroups have factors that favor either virtual or in-person care. Such details can potentially inform and shape public policy surrounding the implementation and utilization of virtual care across various jurisdictions. Therefore, building upon our previously published work, we aimed to compare physician specialty-specific outcomes between virtual care ambulatory visits and in-person care. Specifically, the main objective of our study was to compare subsequent ambulatory visits, hospital and ICU admissions, surgeries, and mortality at 7- and 30-day between patients with initial virtual care visits and in-person visits across different specialities.

## Methods

In this retrospective observational study, we explored ambulatory health care visits captured in administrative databases held at ICES, formerly known as the Institute for Clinical Evaluative Sciences, within the province of Ontario. We focused on virtual care visits and in-person ambulatory care visits occurring during the SARS-CoV-2 pandemic. The data extraction period spanned from March 14, 2020, when the pandemic was officially declared in Ontario, to March 13, 2022. To ensure the accuracy of our results and prevent the inclusion of subsequent outcomes as new index visits, a washout period of 30 days was implemented for each visit. We excluded index visits associated with outpatient surgery, chemotherapy, radiation, or COVID vaccinations based on billing data. In cases where multiple outpatient visits were billed on the same day, we carefully examined the distribution of specialties for those visits. If primary care or emergency medicine (EM) visits were recorded on such days, only the primary care or EM visits were retained for our analysis. Records that lacked primary care or EM visits or contained both types were excluded from our study. Virtual care visits were identified using billing codes specifically developed for virtual care billing purposes, encompassing all modifications made to the codes as the pandemic progressed. To ensure the selection of appropriate in-person intra-pandemic visits, we conducted a thorough review of the schedule of benefits, utilizing only office-based outpatient billing codes. Patients without a valid Ontario health card were excluded from analysis.

### Data sources

To ascertain physician billings for medically necessary care, we used the provincial Ontario Health Insurance Plan (OHIP) database. The Canadian Institute of Health Information National Ambulatory Care Reporting System (CIHI-NACRS) database was used to identify emergency department (ED) visits, which contains anonymized and abstracted data for all ED visits in Ontario. Information regarding acute care hospitalizations and in-patient surgical care was obtained from CIHI’s Discharge Abstract Database (DAD). The Same Day Surgery databases were utilized to capture outpatient surgeries. Mortality data, including out-of-hospital deaths, for all Ontario residents were obtained from The Registered Persons Database (RPDB). The Resident Assessment Instrument for Home Care (RAI-HC) was used to identify long-term care residents and recipients of homecare. The Immigration, Refugees and Citizenship Canada (IRCC) database was used to identify immigration status. The Ontario Drug Benefit (ODB) database contains medical prescriptions for individuals aged 65 and above, as well as patients with provincial drug coverage. These databases were also used to identify patient comorbidities. Data were linked between databases using unique encoded identifiers and analyzed at ICES. Ontario has universal healthcare coverage for medically necessary care therefore, these databases contain the majority of healthcare utilization data within the province. All data for analysis was extracted in March 2023.

### Population matching

Virtual care visits were matched 1:1 to in-person ambulatory care visits on a visit-to-visit basis, hard-matching on the variables outlined in Table 1. Patients may have been included in the study on multiple occasions if they had multiple visits during the study period. The variables utilized for matching encompassed demographics (age, sex, LHIN, rurality), the Ontario Marginalization Index and its components, long-term care resident status, Johns Hopkins ADG score, and previous healthcare utilization.

Because of the large number of virtual and in-person visits, matching was done on a random 20% sample of visits from each cohort. Additionally, several baseline characteristics of each population were compared after matching, including income quintile, immigration status, frailty, receipt of home care, receipt of ODB, and comorbidities. Balance in baseline covariates between the matched groups was assessed using standardized differences. Standardized differences below 0.10 were considered indicative of well-matched groups.

**Table 1:** Matching variables applied between populations.

Variables	Groupings
Sex (as declared with the Ministry of Health)	M, F
Age (yrs)	0–6, 7–12, 13–17, 18–34, 35–44, 45–54, 55–64, 65–74, 75–84, 85+
Local Health Integration Network (LHIN)	Regions 1 through 14
Rural status	Urban, Rural, and missing
Ontario Marginalization Index (ONMARG) Summary Quintiles	1 through 5, data with missing visits matched to each other across populations
Provincially funded long-term care (LTC) resident	Yes, no
Hopkins Aggregated Diagnosis Groups (ADG) score	0, 1–3, 4–6, 7–10, 11+
Number of hospitalizations in the previous year	0,1,2+
Number of PCP visits in the previous year	0,1–5,6–10, 11–15, 16+, not-rostered
Healthcare provider type	Visit provider specialties were matched to one another directly on a 1:1 basis with the exceptions of the categories presented below: Surgery—(cardiothoracic surgery, general surgery, orthopedic surgery, otolaryngology, plastic surgery, neurosurgery, thoracic surgery, and vascular surgery) Oncology- (medical oncology, and therapeutic radiology) Medicine subspecialties (cardiology, endocrinology, hematology, infectious disease, nephrology, neurology, respiratory disease, and rheumatology) Other (clinical immunology, community medicine, diagnostic radiology, genetics, nuclear medicine, ophthalmology, pathology microbiology and clinical biochemistry, and physical medicine)

### Outcome measures and analyses

The study primarily aimed to assess the incidence of acute revisits for care, hospital admissions, and mortality within 7 and 30 days across OHIP-defined specialty categories. These primary outcomes were derived from a previous study that reported aggregate outcomes [3]. Notably, when evaluating primary care specialties, the outcome measures "revisit to same specialty" and "in-person/virtual visit to primary care" were considered equivalent. Our analysis encompassed a total of eight outcomes:

1. Revisit to same physician/specialty within 7 and 30 days
2. In-person visit to primary care within 7 and 30 days
3. Virtual visit to primary care within 7 and 30 days
4. Hospital admission within 7 and 30 days
5. Surgery within 7 and 30 days
6. ICU admission within 7 and 30 days
7. ED visit within 7 and 30 days
8. Mortality at 7 and 30 days

Subsequent visits were identified by analyzing physician billings in the OHIP database within the specified timeframe. Mortality data was obtained by referencing the RPDB within the designated timeframes. Specialty-specific grouping was conducted based on the groupings defined in Table 1. These groupings were designed to ensure sufficient patient volumes within each group, enabling matching between populations. Specialty types were determined using specialty identifying codes associated with each provider in the OHIP Database. However, it is important to note that in some cases, practitioners may have a practice that differs from their specialty code (e.g., a General Practitioner working in an emergency department). Therefore, only in-office Primary Care billing codes were used to represent in-person care. For virtual care, specialty-specific codes were unavailable during the pandemic, so virtual billing codes were attributed to the specialist code under which they were billed. In cases where dual practice was possible, visits were coded according to physician specialty codes, as disambiguation was not possible.

To compare outcomes between populations, multivariate logistic regression was employed. Odds ratios (ORs) with 99% confidence intervals (CIs) were selected to account for the numerous comparisons performed in the analysis. These results were presented using Forrest plots.

### Ethics and approval

ICES’s collection and use of data is authorized under Section 45 of Ontario’s Personal Health Information Protection Act (PHIPA) as a prescribed entity, which is exempt from review by a Research Ethics Board[4,5]. The use of the data in this study is authorized under section 45 and approved by ICES’s Privacy and Legal Office.

## Results

### Population matching

After exclusions, there were 47,092,297 visits in the virtual care visits and 46,270,897 visits in the in-person care visits who were eligible for matching. Table 2 outlines the exclusion criteria applied as well as the quantitative results of these exclusions. A total of 9,340,519 patients were matched within each population. Patient visits required a valid health insurance number and billing data on the visit which included provider specialty. Multiple visits on a single day were also excluded based on the previously mentioned strategy.

**Table 2:** Population exclusion criteria applied to cohorts.

Inclusion/Exclusion	Included	Excluded
**Total population 1 (virtual care visits) pre-exclusions**	**91,801,929**	
Unique on IKN, record date, and specialty	76,771,554	15,030,375
Unique on IKN and record date (specialty exclusions)	76,051,175	720,379
30-day washout period	47,203,634	28,847,541
**Total population 2 (in-person visits) pre-exclusions**	**81,543,934**	
Unique on IKN, record date, and specialty	81,326,940	216,994
Unique on IKN and record date (specialty exclusions)	78,017,303	3,309,637
30-day washout period	46,421,792	31,595,511
**Combined populations**	**95,032,425**	
Non-Ontario postal code on service date, or death date occurring prior to service date	94,767,868	264,557
**Full cohort after above exclusions**	**94,767,868**	
Virtual care population	47,092,297	111,337
Population 1 20% subsample	9,418,460	37,673,837
In-person care population	46,270,897	150,895
**Matched cohort populations**		
Virtual care visits	9,340,519	77,941
In-person visits	9,340,519	36,930,378

Excellent matching results were achieved between populations. A full description of this match is available within our previous published manuscript [3]. Of the matched sample, a total of 6,592,845 visits in each cohort were conducted by physicians with family practice billing codes, representing 70.6% of the total sample. Aside from the general practitioner specialty, medical subspecialties (8.5%), psychiatry (3.5%), pediatrics (3.4%) and surgery (3.2%) represent the 5 specialty groupings with the highest overall virtual care usage.

### Patient outcomes

**Fig 1:**
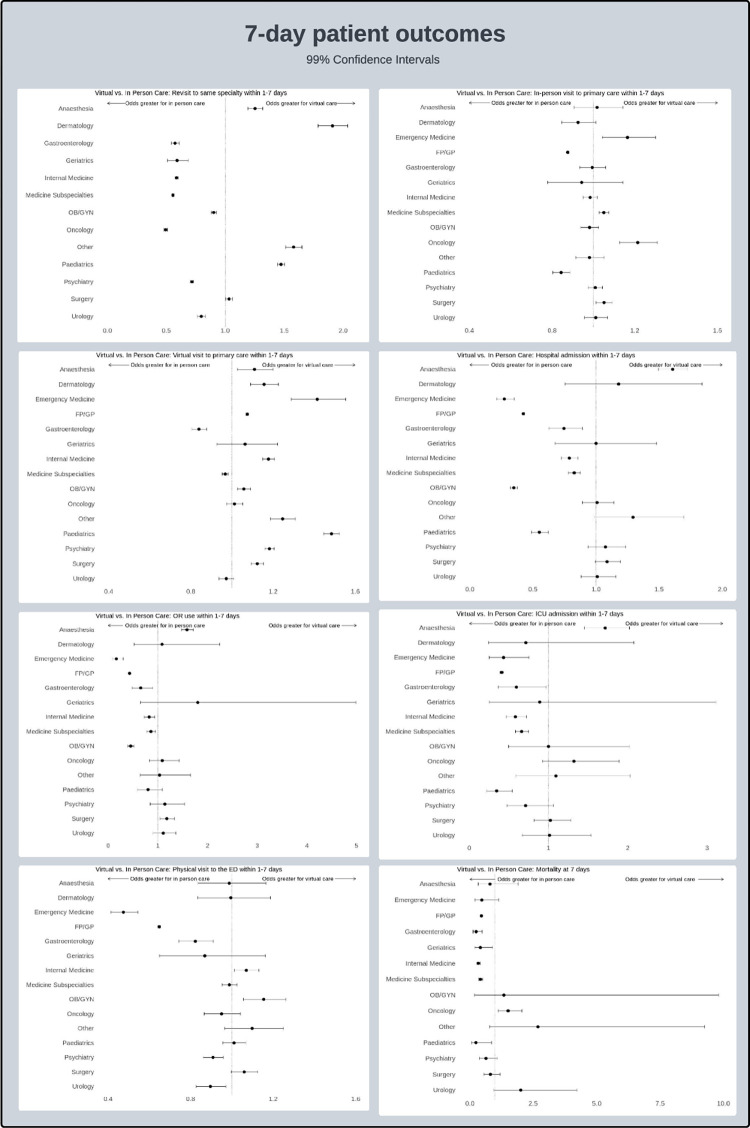
Forrest plots for specialty-specific 7-day outcomes.

**Fig 2:**
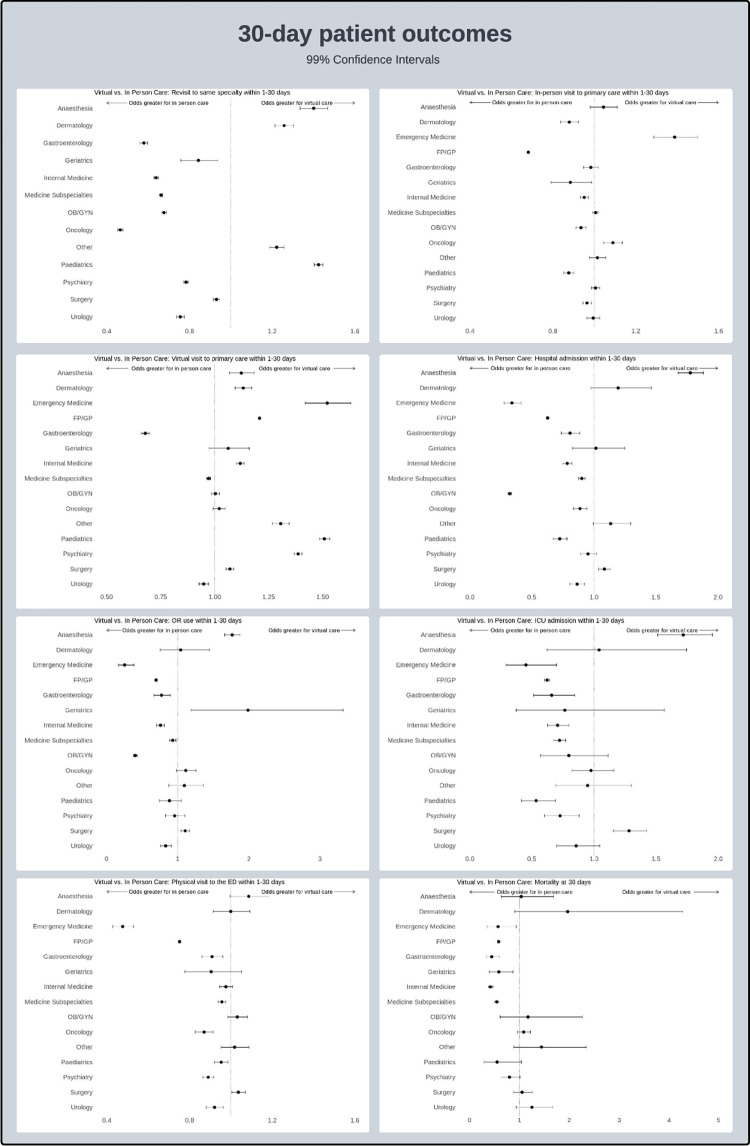
Forrest plots for specialty-specific 30-day outcomes.

An analysis of the same outcome measures between the defined populations is summarized in Tables 3 and 4. Forrest plots are demonstrated in Figs 1 and 2. Overall differences in patterns of outcomes based on specialty are visible within the data although overall trends are relatively similar.

**Table 3:** Specialty-specific outcome comparisons between virtual care visits relative to in-person care visits at 7 days.

Specialty Category	Outcome	Virtual Care	In-Person visits	Total	Standardized Difference	P-value	OR**	Lower	Upper
Anaesthesia	**N**	**N = 24,818**	**N = 24,818**	**N = 49,636**					
	Revisit to same specialty within 1–7 days	8,974 (36.16%)	7,768 (31.30%)	16,742 (33.73%)	**0.10**	**< .001**	**1.253**	**1.192**	**1.317**
	In-person visit to primary care within 1–7 days	1,073 (4.32%)	1,055 (4.25%)	2,128 (4.29%)	0.00	0.690	1.018	0.907	1.142
	Virtual visit to primary care within 1–7 days	2,609 (10.51%)	2,380 (9.59%)	4,989 (10.05%)	0.03	**< .001**	**1.110**	**1.027**	**1.200**
	Hospital admission within 1–7 days	3,799 (15.31%)	2,534 (10.21%)	6,333 (12.76%)	**0.15**	**< .001**	**1.607**	**1.496**	**1.725**
	OR use within 1–7 days	3,429 (13.82%)	2,296 (9.25%)	5,725 (11.53%)	**0.14**	**< .001**	**1.588**	**1.474**	**1.710**
	ICU admission within 1–7 days	675 (2.72%)	401 (1.62%)	1,076 (2.17%)	0.08	**< .001**	**1.713**	**1.453**	**2.020**
	Physical visit to the ED within 1–7 days	490 (1.97%)	496 (2.00%)	986 (1.99%)	0.00	0.847	0.988	0.836	1.166
	Mortality at 7 days	16 (0.06%)	20 (0.08%)	36 (0.07%)	0.01	0.505	0.796	0.332	1.907
Dermatology	**N**	**N = 67,250**	**N = 67,250**	**N = 134,500**					
	Revisit to same specialty within 1–7 days	4,564 (6.79%)	2,476 (3.68%)	7,040 (5.23%)	**0.14**	**< .001**	**1.909**	**1.787**	**2.040**
	In-person visit to primary care within 1–7 days	1,682 (2.50%)	1,811 (2.69%)	3,493 (2.60%)	0.01	**0.027**	0.926	0.847	1.012
	Virtual visit to primary care within 1–7 days	4,513 (6.71%)	3,948 (5.87%)	8,461 (6.29%)	0.03	**< .001**	**1.157**	**1.091**	**1.226**
	Hospital admission within 1–7 days	73 (0.11%)	62 (0.09%)	135 (0.10%)	0.01	0.344	1.179	0.754	1.841
	OR use within 1–7 days	26 (0.04%)	24 (0.04%)	50 (0.04%)	0.00	0.777	1.083	0.522	2.248
	ICU admission within 1–7 days	10 (0.01%)	14 (0.02%)	24 (0.02%)	0.00	0.414	0.713	0.245	2.078
	Physical visit to the ED within 1–7 days	432 (0.64%)	434 (0.65%)	866 (0.64%)	0.00	0.946	0.995	0.835	1.187
	Mortality at 7 days	*3–7	*1–5	8 (0.01%)	0.01	**0.034**	7.271	0.456	115.937
Emergency Medicine	**N**	**N = 13,475**	**N = 13,475**	**N = 26,950**					
	In-person visit to primary care within 1–7 days	1,320 (9.80%)	1,154 (8.56%)	2,474 (9.18%)	0.04	**< .001**	**1.165**	**1.043**	**1.302**
	Virtual visit to primary care within 1–7 days	2,098 (15.57%)	1,565 (11.61%)	3,663 (13.59%)	**0.12**	**< .001**	**1.416**	**1.289**	**1.555**
	Hospital admission within 1–7 days	139 (1.03%)	476 (3.53%)	615 (2.28%)	**0.17**	**< .001**	**0.273**	**0.212**	**0.352**
	OR use within 1–7 days	21 (0.16%)	126 (0.94%)	147 (0.55%)	**0.11**	**< .001**	**0.163**	**0.089**	**0.301**
	ICU admission within 1–7 days	32 (0.24%)	73 (0.54%)	105 (0.39%)	0.05	**< .001**	**0.433**	**0.250**	**0.750**
	Physical visit to the ED within 1–7 days	571 (4.24%)	1,136 (8.43%)	1,707 (6.33%)	**0.17**	**< .001**	**0.475**	**0.414**	**0.544**
	Mortality at 7 days	13 (0.10%)	27 (0.20%)	40 (0.15%)	0.03	**0.027**	0.472	0.196	1.138
FP/GP	**N**	**N = 6,592,845**	**N = 6,592,845**	**N = 13,185,690**					
	In-person visit to primary care within 1–7 days	470,810 (7.14%)	531,119 (8.06%)	1,001,929 (7.60%)	0.03	**< .001**	**0.876**	**0.871**	**0.881**
	Virtual visit to primary care within 1–7 days	533,475 (8.09%)	499,715 (7.58%)	1,033,190 (7.84%)	0.02	**< .001**	**1.075**	**1.069**	**1.081**
	Hospital admission within 1–7 days	23,670 (0.36%)	54,973 (0.83%)	78,643 (0.60%)	0.06	**< .001**	**0.424**	**0.416**	**0.433**
	OR use within 1–7 days	5,805 (0.09%)	13,460 (0.20%)	19,265 (0.15%)	0.03	**< .001**	**0.430**	**0.413**	**0.448**
	ICU admission within 1–7 days	3,150 (0.05%)	7,674 (0.12%)	10,824 (0.08%)	0.02	**< .001**	**0.410**	**0.388**	**0.433**
	Physical visit to the ED within 1–7 days	114,850 (1.74%)	174,889 (2.65%)	289,739 (2.20%)	0.06	**< .001**	**0.649**	**0.642**	**0.655**
	Mortality at 7 days	2,862 (0.04%)	6,198 (0.09%)	9,060 (0.07%)	0.02	**< .001**	**0.455**	**0.429**	**0.483**
Gastroenterology	**N**	**N = 101,802**	**N = 101,802**	**N = 203,604**					
	Revisit to same specialty within 1–7 days	3,084 (3.03%)	5,189 (5.10%)	8,273 (4.06%)	**0.10**	**< .001**	**0.574**	**0.541**	**0.610**
	In-person visit to primary care within 1–7 days	3,447 (3.39%)	3,464 (3.40%)	6,911 (3.39%)	0.00	0.835	0.995	0.934	1.060
	Virtual visit to primary care within 1–7 days	7,218 (7.09%)	8,446 (8.30%)	15,664 (7.69%)	0.05	**< .001**	**0.840**	**0.804**	**0.877**
	Hospital admission within 1–7 days	375 (0.37%)	500 (0.49%)	875 (0.43%)	0.02	**< .001**	**0.746**	**0.625**	**0.891**
	OR use within 1–7 days	109 (0.11%)	166 (0.16%)	275 (0.14%)	0.02	**< .001**	**0.655**	**0.477**	**0.901**
	ICU admission within 1–7 days	44 (0.04%)	74 (0.07%)	118 (0.06%)	0.01	**0.006**	**0.593**	**0.363**	**0.970**
	Physical visit to the ED within 1–7 days	1,192 (1.17%)	1,442 (1.42%)	2,634 (1.29%)	0.02	**< .001**	**0.823**	**0.743**	**0.911**
	Mortality at 7 days	18 (0.02%)	72 (0.07%)	90 (0.04%)	0.03	**< .001**	**0.247**	**0.125**	**0.488**
Geriatrics	**N**	**N = 8,812**	**N = 8,812**	**N = 17,624**					
	Revisit to same specialty within 1–7 days	540 (6.13%)	855 (9.70%)	1,395 (7.92%)	**0.13**	**< .001**	**0.591**	**0.508**	**0.688**
	In-person visit to primary care within 1–7 days	376 (4.27%)	397 (4.51%)	773 (4.39%)	0.01	0.440	0.944	0.779	1.143
	Virtual visit to primary care within 1–7 days	792 (8.99%)	749 (8.50%)	1,541 (8.74%)	0.02	0.252	1.064	0.926	1.223
	Hospital admission within 1–7 days	88 (1.00%)	88 (1.00%)	176 (1.00%)	0.00	1.000	1.000	0.675	1.481
	OR use within 1–7 days	18 (0.20%)	10 (0.11%)	28 (0.16%)	0.02	0.130	1.803	0.652	4.987
	ICU admission within 1–7 days	8 (0.09%)	9 (0.10%)	17 (0.10%)	0.00	0.808	0.889	0.254	3.112
	Physical visit to the ED within 1–7 days	150 (1.70%)	172 (1.95%)	322 (1.83%)	0.02	0.216	0.869	0.649	1.163
	Mortality at 7 days	16 (0.18%)	38 (0.43%)	54 (0.31%)	0.05	**0.003**	**0.415**	**0.192**	**0.900**
Internal Medicine	**N**	**N = 272,860**	**N = 272,860**	**N = 545,720**					
	Revisit to same specialty within 1–7 days	24,640 (9.03%)	38,594 (14.14%)	63,234 (11.59%)	**0.16**	**< .001**	**0.587**	**0.574**	**0.601**
	In-person visit to primary care within 1–7 days	12,211 (4.48%)	12,390 (4.54%)	24,601 (4.51%)	0.00	0.243	0.985	0.952	1.019
	Virtual visit to primary care within 1–7 days	27,300 (10.01%)	23,626 (8.66%)	50,926 (9.33%)	0.05	**< .001**	**1.178**	**1.150**	**1.207**
	Hospital admission within 1–7 days	1,760 (0.65%)	2,223 (0.81%)	3,983 (0.73%)	0.02	**< .001**	**0.789**	**0.726**	**0.857**
	OR use within 1–7 days	705 (0.26%)	855 (0.31%)	1,560 (0.29%)	0.01	**< .001**	**0.824**	**0.722**	**0.939**
	ICU admission within 1–7 days	222 (0.08%)	382 (0.14%)	604 (0.11%)	0.02	**< .001**	**0.580**	**0.467**	**0.721**
	Physical visit to the ED within 1–7 days	4,500 (1.65%)	4,211 (1.54%)	8,711 (1.60%)	0.01	**0.002**	**1.070**	**1.012**	**1.132**
	Mortality at 7 days	215 (0.08%)	636 (0.23%)	851 (0.16%)	0.04	**< .001**	**0.331**	**0.269**	**0.406**
Medicine Subspecialties	**N**	**N = 802,211**	**N = 802,211**	**N = 1,604,422**					
	Revisit to same specialty within 1–7 days	38,698 (4.82%)	66,429 (8.28%)	105,127 (6.55%)	**0.14**	**< .001**	**0.556**	**0.546**	**0.566**
	In-person visit to primary care within 1–7 days	29,287 (3.65%)	27,932 (3.48%)	57,219 (3.57%)	0.01	**< .001**	**1.051**	**1.028**	**1.075**
	Virtual visit to primary care within 1–7 days	63,095 (7.87%)	65,007 (8.10%)	128,102 (7.98%)	0.01	**< .001**	**0.967**	**0.953**	**0.982**
	Hospital admission within 1–7 days	4,009 (0.50%)	4,837 (0.60%)	8,846 (0.55%)	0.01	**< .001**	**0.827**	**0.782**	**0.874**
	OR use within 1–7 days	1,146 (0.14%)	1,334 (0.17%)	2,480 (0.15%)	0.01	**< .001**	**0.859**	**0.774**	**0.953**
	ICU admission within 1–7 days	703 (0.09%)	1,065 (0.13%)	1,768 (0.11%)	0.01	**< .001**	**0.659**	**0.582**	**0.747**
	Physical visit to the ED within 1–7 days	9,852 (1.23%)	9,967 (1.24%)	19,819 (1.24%)	0.00	0.411	0.988	0.952	1.025
	Mortality at 7 days	304 (0.04%)	714 (0.09%)	1,018 (0.06%)	0.02	**< .001**	**0.424**	**0.355**	**0.506**
OB/GYN	**N**	**N = 196,273**	**N = 196,273**	**N = 392,546**					
	Revisit to same specialty within 1–7 days	26,967 (13.74%)	29,401 (14.98%)	56,368 (14.36%)	0.04	**< .001**	**0.901**	**0.880**	**0.923**
	In-person visit to primary care within 1–7 days	7,390 (3.77%)	7,517 (3.83%)	14,907 (3.80%)	0.00	0.289	0.981	0.939	1.026
	Virtual visit to primary care within 1–7 days	17,036 (8.68%)	16,196 (8.25%)	33,232 (8.47%)	0.02	**< .001**	**1.058**	**1.027**	**1.090**
	Hospital admission within 1–7 days	1,364 (0.69%)	3,839 (1.96%)	5,203 (1.33%)	**0.11**	**< .001**	**0.348**	**0.321**	**0.378**
	OR use within 1–7 days	555 (0.28%)	1,222 (0.62%)	1,777 (0.45%)	0.05	**< .001**	**0.452**	**0.396**	**0.516**
	ICU admission within 1–7 days	27 (0.01%)	27 (0.01%)	54 (0.01%)	0.00	1.000	1.000	0.496	2.017
	Physical visit to the ED within 1–7 days	1,871 (0.95%)	1,624 (0.83%)	3,495 (0.89%)	0.01	**< .001**	**1.154**	**1.057**	**1.261**
	Mortality at 7 days	*1–5	*1–5	7 (0.00%)	0.00	0.705	1.345	0.185	9.805
Oncology	**N**	**N = 81,472**	**N = 81,472**	**N = 162,944**					
	Revisit to same specialty within 1–7 days	18,083 (22.20%)	29,370 (36.05%)	47,453 (29.12%)	**0.31**	**< .001**	**0.495**	**0.480**	**0.509**
	In-person visit to primary care within 1–7 days	2,686 (3.30%)	2,227 (2.73%)	4,913 (3.02%)	0.03	**< .001**	**1.215**	**1.127**	**1.310**
	Virtual visit to primary care within 1–7 days	10,002 (12.28%)	9,893 (12.14%)	19,895 (12.21%)	0.00	0.410	1.013	0.974	1.054
	Hospital admission within 1–7 days	886 (1.09%)	879 (1.08%)	1,765 (1.08%)	0.00	0.867	1.008	0.891	1.141
	OR use within 1–7 days	187 (0.23%)	172 (0.21%)	359 (0.22%)	0.00	0.428	1.088	0.828	1.428
	ICU admission within 1–7 days	120 (0.15%)	91 (0.11%)	211 (0.13%)	0.01	**0.046**	1.320	0.922	1.888
	Physical visit to the ED within 1–7 days	1,573 (1.93%)	1,653 (2.03%)	3,226 (1.98%)	0.01	0.155	0.950	0.866	1.042
	Mortality at 7 days	179 (0.22%)	119 (0.15%)	298 (0.18%)	0.02	**< .001**	**1.509**	**1.111**	**2.049**
Other	**N**	**N = 91,797**	**N = 91,797**	**N = 183,594**					
	Revisit to same specialty within 1–7 days	9,420 (10.26%)	6,227 (6.78%)	15,647 (8.52%)	**0.12**	**< .001**	**1.580**	**1.512**	**1.651**
	In-person visit to primary care within 1–7 days	2,839 (3.09%)	2,891 (3.15%)	5,730 (3.12%)	0.00	0.485	0.981	0.915	1.052
	Virtual visit to primary care within 1–7 days	6,874 (7.49%)	5,624 (6.13%)	12,498 (6.81%)	0.05	**< .001**	**1.247**	**1.188**	**1.309**
	Hospital admission within 1–7 days	209 (0.23%)	162 (0.18%)	371 (0.20%)	0.01	**0.015**	1.293	0.986	1.695
	OR use within 1–7 days	61 (0.07%)	59 (0.06%)	120 (0.07%)	0.00	0.855	1.034	0.646	1.656
	ICU admission within 1–7 days	36 (0.04%)	33 (0.04%)	69 (0.04%)	0.00	0.718	1.091	0.586	2.031
	Physical visit to the ED within 1–7 days	839 (0.91%)	765 (0.83%)	1,604 (0.87%)	0.01	0.063	1.098	0.965	1.250
	Mortality at 7 days	16 (0.02%)	6 (0.01%)	22 (0.01%)	0.01	**0.033**	2.685	0.779	9.247
Paediatrics	**N**	**N = 318,402**	**N = 318,402**	**N = 636,804**					
	Revisit to same specialty within 1–7 days	45,259 (14.21%)	32,602 (10.24%)	77,861 (12.23%)	**0.12**	**< .001**	**1.473**	**1.444**	**1.504**
	In-person visit to primary care within 1–7 days	5,353 (1.68%)	6,301 (1.98%)	11,654 (1.83%)	0.02	**< .001**	**0.845**	**0.804**	**0.887**
	Virtual visit to primary care within 1–7 days	26,296 (8.26%)	18,309 (5.75%)	44,605 (7.00%)	**0.10**	**< .001**	**1.485**	**1.447**	**1.524**
	Hospital admission within 1–7 days	719 (0.23%)	1,300 (0.41%)	2,019 (0.32%)	0.03	**< .001**	**0.550**	**0.488**	**0.620**
	OR use within 1–7 days	129 (0.04%)	161 (0.05%)	290 (0.05%)	0.00	0.060	0.801	0.591	1.086
	ICU admission within 1–7 days	45 (0.01%)	130 (0.04%)	175 (0.03%)	0.02	**< .001**	**0.345**	**0.221**	**0.539**
	Physical visit to the ED within 1–7 days	4,515 (1.42%)	4,468 (1.40%)	8,983 (1.41%)	0.00	0.617	1.011	0.957	1.068
	Mortality at 7 days	*1–5	*21–25	26 (0.00%)	0.01	**0.002**	**0.238**	**0.066**	**0.857**
Psychiatry	**N**	**N = 327,928**	**N = 327,928**	**N = 655,856**					
	Revisit to same specialty within 1–7 days	42,183 (12.86%)	55,471 (16.92%)	97,654 (14.89%)	**0.11**	**< .001**	**0.717**	**0.704**	**0.730**
	In-person visit to primary care within 1–7 days	12,387 (3.78%)	12,275 (3.74%)	24,662 (3.76%)	0.00	0.467	1.010	0.976	1.044
	Virtual visit to primary care within 1–7 days	49,436 (15.08%)	42,997 (13.11%)	92,433 (14.09%)	0.06	**< .001**	**1.183**	**1.161**	**1.205**
	Hospital admission within 1–7 days	724 (0.22%)	674 (0.21%)	1,398 (0.21%)	0.00	0.181	1.075	0.936	1.235
	OR use within 1–7 days	156 (0.05%)	137 (0.04%)	293 (0.04%)	0.00	0.267	1.139	0.842	1.540
	ICU admission within 1–7 days	71 (0.02%)	100 (0.03%)	171 (0.03%)	0.01	**0.027**	0.710	0.476	1.059
	Physical visit to the ED within 1–7 days	4,599 (1.40%)	5,049 (1.54%)	9,648 (1.47%)	0.01	**< .001**	**0.909**	**0.862**	**0.958**
	Mortality at 7 days	39 (0.01%)	61 (0.02%)	100 (0.02%)	0.01	**0.028**	0.637	0.375	1.083
Surgery	**N**	**N = 302,991**	**N = 302,991**	**N = 605,982**					
	Revisit to same specialty within 1–7 days	18,524 (6.11%)	17,986 (5.94%)	36,510 (6.02%)	0.01	**0.004**	**1.032**	**1.004**	**1.061**
	In-person visit to primary care within 1–7 days	10,334 (3.41%)	9,846 (3.25%)	20,180 (3.33%)	0.01	**< .001**	**1.052**	**1.014**	**1.092**
	Virtual visit to primary care within 1–7 days	22,011 (7.26%)	19,797 (6.53%)	41,808 (6.90%)	0.03	**< .001**	**1.124**	**1.094**	**1.154**
	Hospital admission within 1–7 days	1,674 (0.55%)	1,541 (0.51%)	3,215 (0.53%)	0.01	**0.019**	1.087	0.992	1.191
	OR use within 1–7 days	986 (0.33%)	836 (0.28%)	1,822 (0.30%)	0.01	**< .001**	**1.180**	**1.045**	**1.333**
	ICU admission within 1–7 days	262 (0.09%)	256 (0.08%)	518 (0.09%)	0.00	0.792	1.023	0.816	1.284
	Physical visit to the ED within 1–7 days	3,851 (1.27%)	3,637 (1.20%)	7,488 (1.24%)	0.01	**0.013**	**1.060**	**0.998**	**1.126**
	Mortality at 7 days	79 (0.03%)	97 (0.03%)	176 (0.03%)	0.00	**0.175**	0.814	0.550	1.203
Urology	**N**	**N = 137,583**	**N = 137,583**	**N = 275,166**					
	Revisit to same specialty within 1–7 days	7,648 (5.56%)	9,447 (6.87%)	17,095 (6.21%)	0.05	**< .001**	**0.797**	**0.764**	**0.830**
	In-person visit to primary care within 1–7 days	4,628 (3.36%)	4,577 (3.33%)	9,205 (3.35%)	0.00	0.589	1.012	0.958	1.069
	Virtual visit to primary care within 1–7 days	10,165 (7.39%)	10,422 (7.58%)	20,587 (7.48%)	0.01	0.063	0.973	0.937	1.010
	Hospital admission within 1–7 days	722 (0.52%)	715 (0.52%)	1,437 (0.52%)	0.00	0.853	1.010	0.881	1.158
	OR use within 1–7 days	331 (0.24%)	299 (0.22%)	630 (0.23%)	0.00	0.202	1.107	0.901	1.361
	ICU admission within 1–7 days	77 (0.06%)	76 (0.06%)	153 (0.06%)	0.00	0.936	1.013	0.668	1.537
	Physical visit to the ED within 1–7 days	2,011 (1.46%)	2,239 (1.63%)	4,250 (1.54%)	0.01	**< .001**	**0.896**	**0.827**	**0.970**
	Mortality at 7 days	36 (0.03%)	18 (0.01%)	54 (0.02%)	0.01	**0.014**	2.005	0.952	4.222

*Bolded values show significant differences between populations (standardized difference ≥ 0.1, p-value < 0.05)

**Odds ratio for population 1 (reference = population 3), adjusted for the 8 matching variables other than specialty category

**Table 4:** Specialty-specific outcome comparisons between virtual care visits relative to in-person care visits at 30 days.

Specialty Category	Outcome	Virtual Care	In-Person Care	Total	Standardized Difference	P-value	OR	Lower	Upper
Anaesthesia	**N**	**N = 24,818**	**N = 24,818**	**N = 49,636**					
	Revisit to same specialty within 1–30 days	15,548 (62.65%)	13,583 (54.73%)	29,131 (58.69%)	**0.16**	**< .001**	**1.402**	**1.336**	**1.470**
	In-person visit to primary care within 1–30 days	4,320 (17.41%)	4,173 (16.81%)	8,493 (17.11%)	0.02	0.080	1.044	0.981	1.112
	Virtual visit to primary care within 1–30 days	8,018 (32.31%)	7,431 (29.94%)	15,449 (31.12%)	0.05	**< .001**	**1.124**	**1.067**	**1.183**
	Hospital admission within 1–30 days	6,729 (27.11%)	4,362 (17.58%)	11,091 (22.34%)	**0.23**	**< .001**	**1.777**	**1.678**	**1.883**
	OR use within 1–30 days	6,010 (24.22%)	3,860 (15.55%)	9,870 (19.88%)	**0.22**	**< .001**	**1.765**	**1.662**	**1.874**
	ICU admission within 1–30 days	1,147 (4.62%)	687 (2.77%)	1,834 (3.69%)	**0.10**	**< .001**	**1.719**	**1.513**	**1.953**
	Physical visit to the ED within 1–30 days	2,067 (8.33%)	1,918 (7.73%)	3,985 (8.03%)	0.02	**0.014**	1.086	0.997	1.184
	Mortality at 30 days	58 (0.23%)	56 (0.23%)	114 (0.23%)	0.00	0.851	1.036	0.637	1.688
Dermatology	**N**	**N = 67,250**	**N = 67,250**	**N = 134,500**					
	Revisit to same specialty within 1–30 days	13,904 (20.68%)	11,553 (17.18%)	25,457 (18.93%)	0.09	**< .001**	**1.258**	**1.214**	**1.305**
	In-person visit to primary care within 1–30 days	6,019 (8.95%)	6,735 (10.01%)	12,754 (9.48%)	0.04	**< .001**	**0.879**	**0.837**	**0.923**
	Virtual visit to primary care within 1–30 days	15,685 (23.32%)	14,310 (21.28%)	29,995 (22.30%)	0.05	**< .001**	**1.132**	**1.094**	**1.172**
	Hospital admission within 1–30 days	363 (0.54%)	305 (0.45%)	668 (0.50%)	0.01	**0.024**	1.195	0.976	1.463
	OR use within 1–30 days	128 (0.19%)	123 (0.18%)	251 (0.19%)	0.00	0.752	1.041	0.751	1.442
	ICU admission within 1–30 days	51 (0.08%)	49 (0.07%)	100 (0.07%)	0.00	0.841	1.041	0.621	1.745
	Physical visit to the ED within 1–30 days	1,765 (2.62%)	1,766 (2.63%)	3,531 (2.63%)	0.00	0.986	0.999	0.915	1.092
	Mortality at 30 days	33 (0.05%)	17 (0.03%)	50 (0.04%)	0.01	**0.024**	1.970	0.906	4.286
Emergency Medicine	**N**	**N = 13,475**	**N = 13,475**	**N = 26,950**					
	In-person visit to primary care within 1–30 days	3,916 (29.06%)	3,156 (23.42%)	7,072 (26.24%)	**0.13**	**< .001**	**1.388**	**1.286**	**1.497**
	Virtual visit to primary care within 1–30 days	5,025 (37.29%)	3,874 (28.75%)	8,899 (33.02%)	**0.18**	**< .001**	**1.521**	**1.419**	**1.631**
	Hospital admission within 1–30 days	249 (1.85%)	675 (5.01%)	924 (3.43%)	**0.17**	**< .001**	**0.338**	**0.277**	**0.412**
	OR use within 1–30 days	46 (0.34%)	179 (1.33%)	225 (0.83%)	**0.11**	**< .001**	**0.251**	**0.164**	**0.386**
	ICU admission within 1–30 days	52 (0.39%)	113 (0.84%)	165 (0.61%)	0.06	**< .001**	**0.453**	**0.293**	**0.700**
	Physical visit to the ED within 1–30 days	1,080 (8.01%)	2,038 (15.12%)	3,118 (11.57%)	**0.22**	**< .001**	**0.477**	**0.429**	**0.529**
	Mortality at 30 days	46 (0.34%)	78 (0.58%)	124 (0.46%)	0.04	**0.004**	**0.570**	**0.348**	**0.934**
FP/GP	**N**	**N = 6,592,845**	**N = 6,592,845**	**N = 13,185,690**					
	In-person visit to primary care within 1–30 days	1,171,644 (17.77%)	1,568,100 (23.78%)	2,739,744 (20.78%)	**0.15**	**< .001**	**0.682**	**0.680**	**0.685**
	Virtual visit to primary care within 1–30 days	1,628,090 (24.69%)	1,419,380 (21.53%)	3,047,470 (23.11%)	0.08	**< .001**	**1.208**	**1.204**	**1.212**
	Hospital admission within 1–30 days	69,651 (1.06%)	109,047 (1.65%)	178,698 (1.36%)	0.05	**< .001**	**0.626**	**0.618**	**0.634**
	OR use within 1–30 days	21,071 (0.32%)	30,266 (0.46%)	51,337 (0.39%)	0.02	**< .001**	**0.694**	**0.678**	**0.711**
	ICU admission within 1–30 days	9,738 (0.15%)	15,590 (0.24%)	25,328 (0.19%)	0.02	**< .001**	**0.623**	**0.602**	**0.644**
	Physical visit to the ED within 1–30 days	309,826 (4.70%)	403,425 (6.12%)	713,251 (5.41%)	0.06	**< .001**	**0.752**	**0.747**	**0.756**
	Mortality at 30 days	10,551 (0.16%)	17,746 (0.27%)	28,297 (0.21%)	0.02	**< .001**	**0.581**	**0.563**	**0.601**
Gastroenterology	**N**	**N = 101,802**	**N = 101,802**	**N = 203,604**					
	Revisit to same specialty within 1–30 days	13,501 (13.26%)	21,039 (20.67%)	34,540 (16.96%)	**0.20**	**< .001**	**0.580**	**0.562**	**0.599**
	In-person visit to primary care within 1–30 days	12,099 (11.88%)	12,269 (12.05%)	24,368 (11.97%)	0.01	0.246	0.984	0.949	1.020
	Virtual visit to primary care within 1–30 days	23,918 (23.49%)	31,178 (30.63%)	55,096 (27.06%)	**0.16**	**< .001**	**0.679**	**0.661**	**0.697**
	Hospital admission within 1–30 days	1,464 (1.44%)	1,792 (1.76%)	3,256 (1.60%)	0.03	**< .001**	**0.808**	**0.736**	**0.887**
	OR use within 1–30 days	536 (0.53%)	692 (0.68%)	1,228 (0.60%)	0.02	**< .001**	**0.772**	**0.665**	**0.896**
	ICU admission within 1–30 days	179 (0.18%)	270 (0.27%)	449 (0.22%)	0.02	**< .001**	**0.660**	**0.514**	**0.847**
	Physical visit to the ED within 1–30 days	4,466 (4.39%)	4,873 (4.79%)	9,339 (4.59%)	0.02	**< .001**	**0.910**	**0.861**	**0.962**
	Mortality at 30 days	121 (0.12%)	266 (0.26%)	387 (0.19%)	0.03	**< .001**	**0.445**	**0.334**	**0.593**
Geriatrics	**N**	**N = 8,812**	**N = 8,812**	**N = 17,624**					
	Revisit to same specialty within 1–30 days	1,360 (15.43%)	1,559 (17.69%)	2,919 (16.56%)	0.06	**< .001**	**0.844**	**0.758**	**0.939**
	In-person visit to primary care within 1–30 days	1,275 (14.47%)	1,410 (16.00%)	2,685 (15.23%)	0.04	**0.005**	**0.885**	**0.793**	**0.987**
	Virtual visit to primary care within 1–30 days	2,566 (29.12%)	2,461 (27.93%)	5,027 (28.52%)	0.03	0.080	1.063	0.974	1.161
	Hospital admission within 1–30 days	336 (3.81%)	331 (3.76%)	667 (3.78%)	0.00	0.844	1.016	0.827	1.249
	OR use within 1–30 days	75 (0.85%)	38 (0.43%)	113 (0.64%)	0.05	**< .001**	**1.988**	**1.187**	**3.328**
	ICU admission within 1–30 days	23 (0.26%)	30 (0.34%)	53 (0.30%)	0.01	0.336	0.765	0.374	1.566
	Physical visit to the ED within 1–30 days	615 (6.98%)	673 (7.64%)	1,288 (7.31%)	0.03	0.093	0.905	0.778	1.053
	Mortality at 30 days	70 (0.79%)	117 (1.33%)	187 (1.06%)	0.05	**< .001**	**0.585**	**0.393**	**0.871**
Internal Medicine	**N**	**N = 272,860**	**N = 272,860**	**N = 545,720**					
	Revisit to same specialty within 1–30 days	64,385 (23.60%)	87,831 (32.19%)	152,216 (27.89%)	**0.19**	**< .001**	**0.639**	**0.629**	**0.650**
	In-person visit to primary care within 1–30 days	37,625 (13.79%)	39,192 (14.36%)	76,817 (14.08%)	0.02	**< .001**	**0.952**	**0.932**	**0.971**
	Virtual visit to primary care within 1–30 days	79,760 (29.23%)	73,903 (27.08%)	153,663 (28.16%)	0.05	**< .001**	**1.119**	**1.101**	**1.137**
	Hospital admission within 1–30 days	5,763 (2.11%)	7,253 (2.66%)	13,016 (2.39%)	0.04	**< .001**	**0.785**	**0.749**	**0.822**
	OR use within 1–30 days	2,313 (0.85%)	3,047 (1.12%)	5,360 (0.98%)	0.03	**< .001**	**0.756**	**0.704**	**0.812**
	ICU admission within 1–30 days	805 (0.30%)	1,136 (0.42%)	1,941 (0.36%)	0.02	**< .001**	**0.706**	**0.627**	**0.796**
	Physical visit to the ED within 1–30 days	14,169 (5.19%)	14,487 (5.31%)	28,656 (5.25%)	0.01	0.054	0.976	0.946	1.008
	Mortality at 30 days	789 (0.29%)	1,808 (0.66%)	2,597 (0.48%)	0.05	**< .001**	**0.418**	**0.373**	**0.468**
Medicine Subspecialties	**N**	**N = 802,211**	**N = 802,211**	**N = 1,604,422**					
	Revisit to same specialty within 1–30 days	124,256 (15.49%)	172,779 (21.54%)	297,035 (18.51%)	**0.16**	**< .001**	**0.662**	**0.655**	**0.669**
	In-person visit to primary care within 1–30 days	99,685 (12.43%)	99,190 (12.36%)	198,875 (12.40%)	0.00	0.236	1.006	0.993	1.019
	Virtual visit to primary care within 1–30 days	210,317 (26.22%)	214,552 (26.75%)	424,869 (26.48%)	0.01	**< .001**	**0.972**	**0.962**	**0.981**
	Hospital admission within 1–30 days	15,667 (1.95%)	17,286 (2.15%)	32,953 (2.05%)	0.01	**< .001**	**0.902**	**0.876**	**0.929**
	OR use within 1–30 days	5,170 (0.64%)	5,570 (0.69%)	10,740 (0.67%)	0.01	**< .001**	**0.927**	**0.882**	**0.975**
	ICU admission within 1–30 days	2,665 (0.33%)	3,678 (0.46%)	6,343 (0.40%)	0.02	**< .001**	**0.722**	**0.676**	**0.771**
	Physical visit to the ED within 1–30 days	36,514 (4.55%)	38,045 (4.74%)	74,559 (4.65%)	0.01	**< .001**	**0.957**	**0.938**	**0.976**
	Mortality at 30 days	1,487 (0.19%)	2,698 (0.34%)	4,185 (0.26%)	0.03	**< .001**	**0.544**	**0.501**	**0.592**
OB/GYN	**N**	**N = 196,273**	**N = 196,273**	**N = 392,546**					
	Revisit to same specialty within 1–30 days	75,293 (38.36%)	92,431 (47.09%)	167,724 (42.73%)	**0.18**	**< .001**	**0.677**	**0.665**	**0.688**
	In-person visit to primary care within 1–30 days	22,687 (11.56%)	23,981 (12.22%)	46,668 (11.89%)	0.02	**< .001**	**0.936**	**0.911**	**0.960**
	Virtual visit to primary care within 1–30 days	53,305 (27.16%)	53,169 (27.09%)	106,474 (27.12%)	0.00	0.625	1.004	0.985	1.023
	Hospital admission within 1–30 days	5,532 (2.82%)	15,816 (8.06%)	21,348 (5.44%)	**0.23**	**< .001**	**0.321**	**0.308**	**0.334**
	OR use within 1–30 days	2,303 (1.17%)	5,624 (2.87%)	7,927 (2.02%)	**0.12**	**< .001**	**0.400**	**0.375**	**0.427**
	ICU admission within 1–30 days	106 (0.05%)	133 (0.07%)	239 (0.06%)	0.01	0.081	0.796	0.569	1.114
	Physical visit to the ED within 1–30 days	6,734 (3.43%)	6,537 (3.33%)	13,271 (3.38%)	0.01	0.082	1.032	0.985	1.080
	Mortality at 30 days	34 (0.02%)	29 (0.01%)	63 (0.02%)	0.00	0.529	1.175	0.610	2.261
Oncology	**N**	**N = 81,472**	**N = 81,472**	**N = 162,944**					
	Revisit to same specialty within 1–30 days	31,140 (38.22%)	45,775 (56.18%)	76,915 (47.20%)	**0.37**	**< .001**	**0.465**	**0.453**	**0.478**
	In-person visit to primary care within 1–30 days	9,390 (11.53%)	8,713 (10.69%)	18,103 (11.11%)	0.03	**< .001**	**1.090**	**1.046**	**1.136**
	Virtual visit to primary care within 1–30 days	32,118 (39.42%)	31,736 (38.95%)	63,854 (39.19%)	0.01	0.053	1.021	0.994	1.049
	Hospital admission within 1–30 days	3,446 (4.23%)	3,851 (4.73%)	7,297 (4.48%)	0.02	**< .001**	**0.887**	**0.833**	**0.944**
	OR use within 1–30 days	937 (1.15%)	844 (1.04%)	1,781 (1.09%)	0.01	**0.027**	1.112	0.983	1.258
	ICU admission within 1–30 days	448 (0.55%)	459 (0.56%)	907 (0.56%)	**0.00**	0.714	0.976	0.822	1.159
	Physical visit to the ED within 1–30 days	5,859 (7.19%)	6,629 (8.14%)	12,488 (7.66%)	0.04	**< .001**	**0.870**	**0.829**	**0.914**
	Mortality at 30 days	1,049 (1.29%)	971 (1.19%)	2,020 (1.24%)	0.01	0.081	1.083	0.964	1.218
Other	**N**	**N = 91,797**	**N = 91,797**	**N = 183,594**					
	Revisit to same specialty within 1–30 days	22,725 (24.76%)	19,528 (21.27%)	42,253 (23.01%)	0.08	**< .001**	**1.222**	**1.187**	**1.258**
	In-person visit to primary care within 1–30 days	10,359 (11.28%)	10,227 (11.14%)	20,586 (11.21%)	0.00	0.329	1.015	0.976	1.056
	Virtual visit to primary care within 1–30 days	22,423 (24.43%)	18,452 (20.10%)	40,875 (22.26%)	**0.10**	**< .001**	**1.306**	**1.267**	**1.345**
	Hospital admission within 1–30 days	820 (0.89%)	725 (0.79%)	1,545 (0.84%)	0.01	**0.015**	1.135	0.993	1.297
	OR use within 1–30 days	288 (0.31%)	264 (0.29%)	552 (0.30%)	0.00	0.306	1.092	0.876	1.361
	ICU admission within 1–30 days	131 (0.14%)	138 (0.15%)	269 (0.15%)	0.00	0.669	0.949	0.692	1.301
	Physical visit to the ED within 1–30 days	3,180 (3.46%)	3,126 (3.41%)	6,306 (3.43%)	0.00	0.489	1.018	0.953	1.088
	Mortality at 30 days	69 (0.08%)	48 (0.05%)	117 (0.06%)	0.01	0.052	1.444	0.887	2.349
Paediatrics	**N**	**N = 318,402**	**N = 318,402**	**N = 636,804**					
	Revisit to same specialty within 1–30 days	109,977 (34.54%)	87,601 (27.51%)	197,578 (31.03%)	**0.15**	**< .001**	**1.424**	**1.404**	**1.445**
	In-person visit to primary care within 1–30 days	17,186 (5.40%)	19,357 (6.08%)	36,543 (5.74%)	0.03	**< .001**	**0.877**	**0.852**	**0.902**
	Virtual visit to primary care within 1–30 days	75,919 (23.84%)	55,451 (17.42%)	131,370 (20.63%)	**0.16**	**< .001**	**1.508**	**1.483**	**1.533**
	Hospital admission within 1–30 days	2,002 (0.63%)	2,739 (0.86%)	4,741 (0.74%)	0.03	**< .001**	**0.724**	**0.671**	**0.782**
	OR use within 1–30 days	417 (0.13%)	472 (0.15%)	889 (0.14%)	0.00	0.065	0.883	0.742	1.050
	ICU admission within 1–30 days	160 (0.05%)	298 (0.09%)	458 (0.07%)	0.02	**< .001**	**0.534**	**0.414**	**0.688**
	Physical visit to the ED within 1–30 days	12,079 (3.79%)	12,637 (3.97%)	24,716 (3.88%)	0.01	**< .001**	**0.953**	**0.922**	**0.986**
	Mortality at 30 days	26 (0.01%)	47 (0.01%)	73 (0.01%)	0.01	**0.014**	0.551	0.293	1.037
Psychiatry	**N**	**N = 327,928**	**N = 327,928**	**N = 655,856**					
	Revisit to same specialty within 1–30 days	133,575 (40.73%)	152,240 (46.42%)	285,815 (43.58%)	**0.11**	**< .001**	**0.783**	**0.773**	**0.794**
	In-person visit to primary care within 1–30 days	39,334 (11.99%)	39,152 (11.94%)	78,486 (11.97%)	0.00	0.489	1.006	0.986	1.026
	Virtual visit to primary care within 1–30 days	154,401 (47.08%)	129,746 (39.57%)	284,147 (43.32%)	**0.15**	**< .001**	**1.386**	**1.368**	**1.405**
	Hospital admission within 1–30 days	2,814 (0.86%)	2,951 (0.90%)	5,765 (0.88%)	0.00	0.070	0.952	0.889	1.020
	OR use within 1–30 days	667 (0.20%)	698 (0.21%)	1,365 (0.21%)	0.00	0.401	0.955	0.831	1.099
	ICU admission within 1–30 days	316 (0.10%)	434 (0.13%)	750 (0.11%)	0.01	**< .001**	**0.727**	**0.600**	**0.880**
	Physical visit to the ED within 1–30 days	16,388 (5.00%)	18,226 (5.56%)	34,614 (5.28%)	0.03	**< .001**	**0.891**	**0.865**	**0.917**
	Mortality at 30 days	216 (0.07%)	269 (0.08%)	485 (0.07%)	0.01	**0.016**	0.800	0.631	1.014
Surgery	**N**	**N = 302,991**	**N = 302,991**	**N = 605,982**					
	Revisit to same specialty within 1–30 days	66,336 (21.89%)	70,114 (23.14%)	136,450 (22.52%)	0.03	**< .001**	**0.931**	**0.916**	**0.945**
	In-person visit to primary care within 1–30 days	35,075 (11.58%)	36,146 (11.93%)	71,221 (11.75%)	0.01	**< .001**	**0.965**	**0.945**	**0.986**
	Virtual visit to primary care within 1–30 days	71,869 (23.72%)	68,398 (22.57%)	140,267 (23.15%)	0.03	**< .001**	**1.071**	**1.054**	**1.088**
	Hospital admission within 1–30 days	7,159 (2.36%)	6,628 (2.19%)	13,787 (2.28%)	0.01	**< .001**	**1.084**	**1.036**	**1.133**
	OR use within 1–30 days	4,651 (1.54%)	4,219 (1.39%)	8,870 (1.46%)	0.01	**< .001**	**1.105**	**1.045**	**1.167**
	ICU admission within 1–30 days	1,384 (0.46%)	1,082 (0.36%)	2,466 (0.41%)	0.02	**< .001**	**1.282**	**1.154**	**1.424**
	Physical visit to the ED within 1–30 days	14,148 (4.67%)	13,679 (4.51%)	27,827 (4.59%)	0.01	**0.004**	**1.037**	**1.004**	**1.071**
	Mortality at 30 days	420 (0.14%)	399 (0.13%)	819 (0.14%)	0.00	0.463	1.053	0.879	1.263
Urology	**N**	**N = 137,583**	**N = 137,583**	**N = 275,166**					
	Revisit to same specialty within 1–30 days	26,961 (19.60%)	33,427 (24.30%)	60,388 (21.95%)	**0.11**	**< .001**	**0.756**	**0.738**	**0.775**
	In-person visit to primary care within 1–30 days	16,025 (11.65%)	16,098 (11.70%)	32,123 (11.67%)	0.00	0.665	0.995	0.964	1.026
	Virtual visit to primary care within 1–30 days	34,637 (25.18%)	35,928 (26.11%)	70,565 (25.64%)	0.02	**< .001**	**0.949**	**0.928**	**0.972**
	Hospital admission within 1–30 days	2,756 (2.00%)	3,172 (2.31%)	5,928 (2.15%)	0.02	**< .001**	**0.864**	**0.807**	**0.925**
	OR use within 1–30 days	1,435 (1.04%)	1,723 (1.25%)	3,158 (1.15%)	0.02	**< .001**	**0.830**	**0.757**	**0.911**
	ICU admission within 1–30 days	307 (0.22%)	358 (0.26%)	665 (0.24%)	0.01	**0.048**	0.857	0.701	1.047
	Physical visit to the ED within 1–30 days	6,768 (4.92%)	7,299 (5.31%)	14,067 (5.11%)	0.02	**< .001**	**0.922**	**0.881**	**0.964**
	Mortality at 30 days	176 (0.13%)	141 (0.10%)	317 (0.12%)	0.01	**0.049**	1.251	0.934	1.677

*Bolded values show significant differences between populations (standardized difference ≥ 0.1, p-value < 0.05)

### Interpretation

#### Outcome comparisons

Within the revisit category, most specialties had an increase in return visits for in-person care visits. As mentioned in previous publications that may be attributable to the fact that patients presenting initially for in-person care may have represented more unwell or acute patients, or otherwise required in-person follow up. The exceptions to this were anesthesia, dermatology, pediatrics and the “other” category where return visits were increased for virtual care. All these results were seen for both the 7 and 30-day outcome timeframes.

Regarding in-person follow up to general practitioners, many specialties demonstrated no significant differences between in-person visits or virtual visits. Visits were more likely to occur in person by their GP after being seen virtually by Emergency Medicine, Medicine subspecialties, Oncology and Surgery in the 7-day outcomes analysis. This was also seen at 30 days with Emergency Medicine and Oncology. Patients were more likely to be seen in person by their GP if seen in person previously by their GP or by pediatrics at 7 days. At 30 days, this in-person cohort grew to include Dermatology, Geriatrics, Internal Medicine, OBGYN and Surgery in addition to Pediatrics and GP. The difference in 7 and 30-day timeframes may be attributable to a time delay which is integral to the booking period for in-person follow ups particularly for GPs.

Patients were more likely to be seen virtually by their GP if they were seen in person by Medicine subspecialties and GI at 7 days which expanded to include Urology at 30 days. Otherwise, OBGYN, Oncology and Geriatrics had no differences in virtual follow-up by GPs. All other specialties had higher likelihoods of being seen in virtual follow up if first seen virtually. This may be indicative of a “streaming” phenomenon whereby patients who made use of virtual care continued to be seen through this modality in most clinical settings.

Hospital admissions, OR use and ICU admissions, were all increased in patients who were seen in person within the 7-day timeframe across all specialties, except for Anesthesia. At the 30-day timeframe, hospital admission and ICU use was increased for virtual patients who were seen by Anesthesia and Surgery. OR use was increased for these same virtual specialties in addition to Geriatrics. The inclusion of both Anesthesia and Surgery in this category may reflect preoperative assessments done virtually before surgeries which required post-operative care in an ICU environment. The cause of significant differences between Geriatrics visits within this category is somewhat less clear.

ED presentations were increased in patients seen virtually by OBGYN, and Internal Medicine within 7 days. At 30 days, this was only true for patients seen by Surgery virtually. Patients with visits done in person had higher subsequent ED presentations within Emergency Medicine, GP, Gastroenterology, Psychiatry and Urology at 7 days with the addition of Gastroenterology, Medicine Subspecialties, Oncology and Pediatrics at 30 days.

Finally, mortality was greater in patients seen virtually by Oncology at 7 days although this dissipated at 30days where no specialties demonstrated increase mortality when seen virtually. Otherwise, mortality was increased when patients were seen in person within GP, GI, Geriatrics, Internal Medicine, Medicine subspecialties, and Pediatrics at 7days. Within the 30-day window, Pediatrics mortality was no longer significant although those seen in person in Emergency Medicine had higher rates of mortality within this timeframe.

### Limitations

This study has multiple limitations. Frist, there were visits that were generated on the same calendar day by the same patient but with different clinicians. Our data does not include time-of-day information; therefore, we were unable to assess the chronological order of these visits. To address this limitation, we tried to retain Emergency Department (ED) or primary care visits that occurred on these days. However, there is a possibility of excluding important data because of these exclusions. Nonetheless, the total number of visits excluded because of this was approximately 1% or less of the final population sizes and therefore unlikely to have significantly changed our results.

As noted in our previous studies, we were unable to match for patient acuity between groups. Although this represents an important match criterion, there are no systematic triaging method for outpatient care in Ontario or Canada except for emergency medicine. Although this remains a methodological limitation, it allows for a pragmatic analysis of non-prescriptive system responses to acuity. Although we have limited evidence, the difference in acuity and the likelihood that more acute patients were streamed to in-person care is the most likely candidate explaining worse outcome for patients seen in-person.

It is also important to mention that short-term outcomes are not to be conflated with those experience by patients over several months or years after they experienced care. Many articles describe delay in diagnosis [6–8] and it is possible that these have led to worse outcomes among specific patient types. Our analysis limits itself to 30 days due to contiguity of data and a relatively short timeframe since the declared end of the pandemic.

## Conclusion

When examining individual specialties and specialty groups, it appears overall trends in short-term outcomes between in-person and virtual visits demonstrates that those seen in person had more severe short-term outcomes. These outcomes are not driven principally by any specific specialty grouping. It is likely that this effect is driven principally by the streaming of more unwell patients towards in-person visits.
