# Correction: Specialty-specific Evaluation of Virtual care Outcomes: A retrospective QUality and safety analysis (S-EVOQUe)

**DOI:** 10.1371/journal.pdig.0000827

**Published:** 2025-04-01

**Authors:** Shawn Mondoux, Frank Battaglia, Anastasia Gayowsky, Natasha Clayton, Cailin Langmann, Paul Miller, Alim Pardhan, Julie Mathews, Alex Drossos, Keerat Grewal

The Acknowledgments section for this article is incorrect. The correct Acknowledgements section is as follows: This study was supported by ICES, which is funded by an annual grant from the Ontario Ministry of Health (MOH) and the Ministry of Long-Term Care (MLTC). This study also received funding from the Juravinski Research Institute and the Graham Farquharson Knowledge Translation Fellowship provided by PSI Foundation (SM). The funders had no role in study design, data collection and analysis, decision to publish, or preparation of the manuscript. SM did receive a salary for protected time from the PSI Foundation. This document used data adapted from the Statistics Canada Postal CodeOM Conversion File, which is based on data licensed from Canada Post Corporation, and/or data adapted from the Ontario Ministry of Health Postal Code Conversion File, which contains data copied under license from Canada Post Corporation and Statistics Canada. Parts of this material are based on data and/or information compiled and provided by CIHI and the Ontario Ministry of Health. This study also makes use of the Immigration Refugees and Citizenship Canada’s Permanent Resident Database. The analyses, conclusions, opinions and statements expressed herein are solely those of the authors and do not reflect those of the funding or data sources; no endorsement is intended or should be inferred.
